# Update in acute respiratory distress syndrome

**DOI:** 10.1186/2052-0492-2-2

**Published:** 2014-01-03

**Authors:** Younsuck Koh

**Affiliations:** Department of Pulmonary and Critical Care Medicine, Asan Medical Center, University of Ulsan College of Medicine, Seoul, 138-736 South Korea

**Keywords:** ARDS, The Berlin definition, Treatment, Prone, ECMO, Review

## Abstract

Acute respiratory distress syndrome (ARDS) is characterized by permeability pulmonary edema and refractory hypoxemia. Recently, the new definition of ARDS has been published, and this definition suggested severity-oriented respiratory treatment by introducing three levels of severity according to PaO_2_/FiO_2_ and positive end-expiratory pressure. Lung-protective ventilation is still the key of better outcome in ARDS. Through randomized trials, short-term use of neuromuscular blockade at initial stage of mechanical ventilation, prone ventilation in severe ARDS, and extracorporeal membrane oxygenation in ARDS with influenza pneumonia showed beneficial efficacy. However, ARDS mortality still remains high. Therefore, early recognition of ARDS modified risk factors and the avoidance of aggravating factors during the patient's hospital stay can help decrease its development. In addition, efficient antifibrotic strategies in late-stage ARDS should be developed to improve the outcome.

## Introduction

Acute respiratory distress syndrome (ARDS) is a permeability pulmonary edema characterized by increased permeability of pulmonary capillary endothelial cells and alveolar epithelial cells, leading to hypoxemia that is refractory to usual oxygen therapy. In a national study in Iceland, the incidence of ARDS almost doubled, but hospital mortality decreased during the 23 years of observation [1]. In a prospective study in Spain, despite use of lung-protective ventilation, overall ICU and hospital mortality of ARDS patients is still higher than 40% [2]. The aim of this review is to provide an update on ARDS.

## Review

### The evolution of the definition of ARDS

The first definition of ARDS dates to Ashbaugh and colleagues in 1967 [3], followed by the American-European Consensus Conference's definition in 1994. The American-European Consensus Conference's definition in 1994, which has been challenged over the years in several studies since the assessment of oxygenation defect, does not require standardized ventilatory support [4]. Recently, a new consensus definition of ARDS, the Berlin definition, has been published [5]. The new definition of ARDS maintains a link to the 1994 definition with diagnostic criteria of timing, chest imaging, origin of edema, and hypoxemia. According to the revised definition of ARDS, a minimum level of positive end-expiratory pressure (PEEP) and mutually exclusive PaO_2_/FiO_2_ thresholds was chosen to differentiate between three levels of severity (mild, moderate, and severe) of ARDS. The revised definition appears to have improved predictive validity for mortality of its spectrum of severity [5]. The revised definition presents a severity-oriented method for respiratory management of ARDS [6]. Histopathological findings have been correlated to severity and duration of ARDS [7]. Using clinical criteria, the revised definition for ARDS allowed for the identification of severe ARDS of more than 72 h as a homogeneous group of patients characterized by a high proportion of diffuse alveolar damage [7].

### Pathogenesis of ARDS

In addition to the classical views of ARDS including the role of cellular and humoral mediators, the role of the renin-angiotensin system (RAS) has been highlighted. The RAS is thought to contribute to the pathophysiology of ARDS by increasing vascular permeability. Angiotensin-converting enzyme (ACE) is a key enzyme of the RAS that converts inactive angiotensin I to the vasoactive and aldosterone-stimulating peptide angiotensin II and also metabolizes kinins along with many other biologically active peptides. ACE is found in varying levels on the surface of lung epithelial and endothelial cells [8]. Angiotensin II induces apoptosis of lung epithelial and endothelial cells and is a potent fibrogenic factor [9]. Based on these biological properties of ACE, there is considerable interest in its potential involvement in acute lung injury (ALI)/ARDS [10, 11].

### Cor pulmonale in ARDS

Since the first publication demonstrating the presence of pulmonary hypertension and elevated pulmonary vascular resistance in patients with severe acute respiratory failure [12], the development of acute cor pulmonale in ARDS has been considered a poor prognostic factor. In two recent prospective observational studies, cor pulmonale occurrence was not negligible (up to one fourth) in ARDS patients ventilated with airway pressure limitations, was associated with sepsis, and was a risk factor for 28-day mortality [13, 14]. Considering these findings together with the association of high PEEP levels and elevated plateau pressure with pulmonary artery pressure [15], careful monitoring of acute cor pulmonale is recommended in ARDS.

### Diagnosis and early intervention

Differential diagnosis between cardiogenic pulmonary edema (CPE) and ARDS is sometimes not easy. The accuracy of the portable chest radiograph to detect pulmonary abnormalities consistent with ARDS is significantly limited [16]. In a study using chest computed tomography, upper-lobe-predominant ground-glass attenuation, central-predominant ground-glass attenuation, and central airspace consolidation were associated with high positive predictive values (95.2%, 92.3%, and 92.0%, respectively) and moderate negative predictive values (47.5%, 51.4%, and 50.0%, respectively) to diagnose CPE [17]. Measurement of the extravascular lung water index and the pulmonary vascular permeability index (PVPI) [18] using a transpulmonary thermodilution method seemed to be a useful quantitative diagnostic tool for ARDS in patients with hypoxemic respiratory failure and radiologic infiltrates. In one study, A PVPI value of 2.6–2.85 provided a definitive diagnosis of ALI/ARDS (specificity, 0.90–0.95), and a value <1.7 ruled out an ALI/ARDS diagnosis (specificity, 0.95) [18].

### Clinical trials of anti-inflammatory therapy

Although ARDS is an acute lung inflammation in which diverse inflammatory cells and mediators are involved, multiple anti-inflammatory interventions have not shown improved survival. Clinical trials using corticosteroids, prostaglandins, nitric oxide, prostacyclin, surfactant, lisofylline, ketoconazole, *N*-acetylcysteine, and fish oil have been unable to show a statistically significant improvement in patient mortality. The use of corticosteroids to attenuate inflammation remains controversial. Moreover, the ARDSnet study of steroid treatment [19] revealed that administration after 14 days of disease onset could be harmful. Up to now, the efficacy of low-dose corticosteroids [20] for the alleviation of inflammation, to reduce organ dysfunction and to improve survival, especially in sepsis associated ARDS, has not been fully elucidated.

### Mechanical ventilation

Numerous lines of evidence have demonstrated that inappropriate mechanical ventilatory settings can produce further lung damage to patients with ARDS. Ventilator-induced lung injury seems to be attributed to end-inspiratory overdistension and a low end-expiratory lung volume, allowing repeated collapse and re-expansion with each respiratory cycle (tidal recruitment). Tidal recruitment results in high shear force on alveolar walls and small airways during inflation, especially at the interfaces between collapsed and aerated alveoli. Therefore, low tidal volume (6 mL/kg of predicted body weight), limitation of plateau pressure (less than 28–30 cm H_2_O), and appropriate PEEP is a key component of a lung-protective ventilatory strategy (LPVS) [21]. Since then, the lung-protective mechanical ventilation strategy has been the standard practice for the management of ARDS. In a retrospective observational study of 104 patients with ARDS caused by pandemic influenza A/H1N1 infection admitted to 28 ICUs in South Korea, low-tidal volume (TV) mechanical ventilation still benefited patients with ARDS caused by viral pneumonia. Patients with TV less than or equal to 7 mL/kg required ventilation, ICU admission, and hospitalization for fewer days than those with TV greater than 7 mL/kg (11.4 vs. 6.1 days for 28-day ventilator-free days, 9.7 vs. 4.9 days for 28-day ICU-free days, and 5.2 vs. 2.4 days for 28-day hospital-free days, respectively). A tidal volume greater than 9 mL/kg (hazard ratio, 2.459; *P* = 0.003) and the Sequential Organ Failure Assessment score (hazard rate, 1.158; *P* = 0.014) were significant predictors of 28-day ICU mortality [22].

The lung-protective ventilation strategy is both safe and potentially beneficial in patients who do not have ARDS at the onset of mechanical ventilation. In mechanically ventilated patients without ARDS at the time of endotracheal intubation, the majority of data favors lower tidal volume to reduce progression to ARDS [23]. Septic patients without ARDS who were ventilated with a protective strategy using a plateau pressure <30 cmH_2_O showed better outcomes and a lower incidence of ARDS than those ventilated without this limit on plateau pressure [24]. A recent meta-analysis also showed that protective ventilation with low tidal volumes was associated better clinical outcomes even in patients without ARDS [25]. The use of very low TV combined with extracorporeal CO_2_ removal has the potential to further reduce ventilator-associated lung injury. Whether this strategy will improve survival in ARDS patients remains to be determined [26].

To select the optimal PEEP level to prevent the undesirable tidal recruitment together with the minimization of alveolar overdistension is not easy. Traditionally, the level of PEEP has been set according to the required level of FiO_2._ Simple elevation of the PEEP level which is more than that of the ARDSnet clinical trial group of low TV was shown to not improve clinical outcome [27]. Another way to set the PEEP level is to employ a decremental PEEP trial after alveolar recruitment maneuvers (ARM). An ARM has the advantage of standardizing the history of lung volume and to let the lung remain more open at the end of expiration. However, the application of early ARM with low tidal volume has not been proved efficacious for the reduction of mortality [28, 29]. The PEEP level could be set according to a level of transpulmonary pressure during expiration. One study demonstrated the efficacy of esophageal pressure-guided PEEP on the improvement of oxygenation and lung compliance in ALI [30]. The researchers set the PEEP at a level to guarantee that transpulmonary pressure during end-expiratory occlusion would stay between 0 and 10 cm H_2_O as well as keep transpulmonary pressure during end-inspiratory occlusion at less than 25 cm H_2_O [30]. A problem with setting the PEEP according to the transpulmonary pressure is the technical difficulty in achieving accurate esophageal pressure using an esophageal balloon catheter [31]. Recently, electrical impedance tomography has been introduced as a true bedside technique, which provides information on regional ventilation distribution [32].

### Prone ventilation

Prone position reduces the transpulmonary pressure gradient, recruiting collapsed regions of the lung without increasing airway pressure or hyperinflation. Prone ventilation showed improved oxygenation and improved outcomes in severe hypoxemic patients with ARDS [33]. Prone ventilation was more effective in obese patients with ARDS than in non-obese ARDS patients [34]. In a study investigating whether there is any interdependence between the effects of PEEP and prone positioning, prone positioning further decreased non-aerated tissue (322 ± 132 to 290 ± 141 g, *P* = 0.028) and reduced tidal hyperinflation observed at PEEP 15 in the supine position (0.57% ± 0.30% to 0.41% ± 0.22%) [35]. Cyclic recruitment/de-recruitment only decreased when high PEEP and prone positioning were applied together (4.1% ± 1.9% to 2.9% ± 0.9%, *P* = 0.003), especially in patients with high lung recruitability [35]. These results showed that prone ventilation decreases alveolar instability and hyperinflation observed at high PEEP in ARDS patients.

### Extracorporeal membrane oxygenation and high-frequency oscillatory ventilation for ARDS

Extracorporeal membrane oxygenation (ECMO) is a therapy that has been used in severe cases of ARDS when patients fail to improve with traditional management. Major technological improvements in ECMO machines and the positive results of the conventional ventilatory support versus extracorporeal membrane oxygenation for severe adult respiratory failure (CESAR) trial [36] have reignited interest in veno-venous ECMO in patients with severe ARDS. Recent literature shows varying mortality rates for the use of ECMO for ARDS. Although transfer of patients to an ECMO center for treatment using specific criteria and indications may improve outcomes, credible evidence supporting a mortality benefit of ECMO is lacking. Further research is needed regarding the timing of the initiation of ECMO, the standardization of therapy and monitoring, and understanding which type of ECMO reduces morbidity and mortality rates in patients with ARDS.

High-frequency oscillatory ventilation (HFOV) seems ideal for lung protection in acute respiratory distress syndrome. HFOV was effective in improving oxygenation in adults with ARDS, particularly when instituted early [37]. Changes in PaO_2_/FiO_2_ during the first 3 h of HFOV helped identify patients that are more likely to survive [37] and showed a promising outcome compared with ARDS patients without current LPVS [38]. In adults with moderate-to-severe ARDS, early application of HFOV compared with an employment of a ventilation strategy of low tidal volume and high positive end-expiratory pressure, does not reduce, and may increase, in-hospital mortality [39].

### Neuromuscular blockade and sedation

Neuromuscular blocking agents (NMBAs) are commonly used in ARDS, but the benefits and the risks of using these agents are controversial. In a recent randomized trial [40], the use of NMBAs in ARDS patients showed a beneficial outcome. In addition, short-term infusion of cisatracurium besylate reduced hospital mortality and barotrauma and did not appear to increase ICU-acquired weakness for critically ill adults with ARDS [40]. The use of alpha-2 adrenergic agonists (e.g., dexmedetomidine) could be used in a non-invasive positive pressure ventilation trial in a select group of ARDS patients, as this class of drugs preserve respiratory drive [41], lower oxygen consumption, and pulmonary hypertension and increase diuresis.

### Fluid management and a bronchodilator use

Because vascular and epithelial permeability is increased in ARDS, fluid management is one of the most difficult measures to manage in septic shock patients with ARDS. A conservative fluid management strategy maintaining a relatively low central venous pressure is associated with the need for fewer days of mechanical ventilation compared with a liberal fluid management strategy in ARDS [42]. However, conservative fluid management is highly recommended after hemodynamic stabilization in ARDS patients. In hemodynamically unstable patients, dynamic monitoring of lung fluid balance needs to be implemented to guide the administration of fluids in ARDS patients [43]. Despite a putative beneficial role in the resolution of alveolar edema seen in preliminary studies, recent evidence has indicated significant detrimental effects associated with beta-2 agonist use in ARDS patients [44].

### Experimental trials

In experimental models of ARDS mesenchymal stem cells (MSCs), transplantation improved the regeneration of lung tissue [45, 46]. The benefits of these MSCs are derived not only from the incorporation of these cells in the damaged lung, but also from their interaction with damaged lung cells and immunologic modulation [47]. MSCs can also control oxidative stress, transfer functional mitochondria to the damaged cells, and control bacterial infection by secreting antibacterial peptides [48]. Most of these studies administered MSCs as a pretreatment, and the use of MSCs is still highly experimental as a treatment or prevention strategy of ARDS.

### Prognosis and quality of life of survivors from ARDS

In a meta-analysis, an insertion/deletion (I/D) polymorphism in the ACE gene was not associated with susceptibility to ALI/ARDS for any genetic model. However, the ACE I/D polymorphism was associated with an increased mortality risk of ALI/ARDS in Asian subjects [49]. After correcting for multiple comparisons, this finding remained significant, and it was shown that the genotype of the I/D polymorphism in ACE may be a predictor of ALI/ARDS mortality in Asian populations [49].

Along with high mortality risks, survivors suffer significant decrements in their quality of life [50]. In survivors of acute lung injury, there was no difference in physical function, survival, or multiple secondary outcomes at 6 and 12 months follow-up after initial trophic or full enteral feeding [51].

## Conclusions

Currently, in spite of the remarkable advancements in the understanding of its pathogenesis, the only effective therapeutic measure to decrease mortality is low-tidal volume mechanical ventilation and prone ventilation for severe ARDS cases. In extreme, life-threatening cases, ECMO seems to serve as a bridge to recovery and enables lung-protective ventilation. Most ARDS patients die of multi-organ failure rather than irreversible respiratory failure, indicating that ARDS is closely associated with other organs by neurological, biochemical, metabolic, and inflammatory reactions. Moreover, the lungs may play an important role in the development of non-pulmonary organ failure in ARDS. Thus, early recognition of ARDS modified risk factors and the avoidance of aggravating factors during the patient's hospital stay (e.g., non-protective mechanical ventilation, multiple blood product transfusions, positive fluid balance, ventilator-associated pneumonia, and gastric aspiration) can help decrease its incidence. In addition, efficient antifibrotic strategies are still lacking for patients with late-stage ARDS. Therefore, new therapies that address the underlying pathophysiology are needed to reduce the mortality of patients with ARDS.
